# Epilepsy, Consciousness and Neurostimulation

**DOI:** 10.3233/BEN-2011-0319

**Published:** 2011-03-29

**Authors:** Manny Bagary

**Affiliations:** Department of NeuropsychiatryThe Barberry BuildingUK Centre for Mental HealthEdgbastonBirminghamUK

## Abstract

Consciousness is often disrupted in epilepsy. This may involve altered responsiveness or changes in awareness of self and subjective experiences. Subcortical arousal systems and paralimbic fronto-parietal association cortices are thought to underpin current concepts of consciousness. The Network Inhibition Hypothesis proposes a common neuroanatomical substrate for impaired consciousness during absence, complex partial and tonic-clonic seizures.

Neurostimulation in epilepsy remains in its infancy with vagal nerve stimulation (VNS) as the only firmly established technique and a series of other methods under investigation including deep brain stimulation (DBS), intracranial cortical stimulation and repetitive transcranial magnetic stimulation (rTMS). Many of these systems impact on the neural systems thought to be involved in consciousness as a continuous duty cycle although some adaptive (seizure triggered) techniques have been developed.

Theoretically, fixed duty cycle neurostimulation could have profound effects on responsiveness, awareness of self and subjective experience. Animal studies suggest vagal nerve stimulation positively influences hippocampal long term potentiation. In humans, a chronic effect of increased alertness in VNS implanted subjects and acute effect on memory consolidation have been reported but convincing data on either improvements or deterioration in attention and memory is lacking. Thalamic deep brain stimulation (DBS) is perhaps the most interesting neurostimulation technique in the context of consciousness. Neither bilateral anterior or centromedian thalamic nucleus DBS seem to affect cognition. Unilateral globus pallidus internus DBS caused transient wakefulness in an anaesthetised individual.

As intracranial neurostimulation, particularly thalamic DBS, becomes more established as a clinical intervention, the effects on consciousness and cognition with variations in stimulus parameters will need to be studied to understand whether these secondary effects of neurostimulation make a significant positive (or adverse) contribution to quality of life.

## 1. Concepts of consciousness

In the absence of a universally accepted definition, those of us working in clinical medicine tend to adopt a pragmatic meaning to the term consciousness to include the degree of wakefulness, the ability to perceive and to interpret sensory information enabling appropriate responses. These concepts concentrate on the responsiveness of an individual to the environment, much of which we can attempt to assess objectively with a range of observations including standardised scales with some prognostic value such as the Glasgow Coma Scale. Less attention is directed to the related concept of consciousness that involves awareness of self to include subjective experiences and the recollection of those subjective experiences.

Consciousness seems to be dependent on the association cortex and subcortical arousal systems in the thalamus, upper brainstem, and basal forebrain [63]. The concept of subjective experience has been related to cortical paralimbic prefrontal and parietal regions including anterior cingulate/medial prefrontal and posterior cingulate/medial parietal cortices connected both directly and through the thalamus. These integrated neural circuits are a common feature in different patterns of regional hemodynamic interaction in different mental states and experiences [9,28,45,46,52]. Episodic memory retrieval is an integral component of temporal self-awareness and consciousness. This has been described as extended self [6] or autonoetic consciousness [26,65] as it involves retrieval of memories of personally experienced events. The related concept of the minimal self involves the subjective sense of owner-

ISSN 0953-4180/11/$27.50 © 2011 – IOS Press and the authors. All rights reserved ship of experiences [25] and seems to involve the same paralimbic structures as autonoetic consciousness [41, 69]. Theoretically impairment of consciousness may occur though disruption of either or both components of these cortical-subcortical systems.

## 2. Consciousness in epilepsy

Historically, Hughlings Jackson considered consciousness in epilepsy to be dependent on the origin and spread of discharge to "higher centres" [35]. Subsequent studies by Penfield and Jasper highlighted the importance of subcortical structures in consciousness. Penfield observed that epileptic patients who had large cortical lesions or corpus callosum lesions suffered minimal impairment of consciousness. However, brain stem pressure was observed to cause immediate, reversible loss of consciousness suggesting the diencephalon and the brain stem were the major structural influences on consciousness with the cortex involved in higher level functioning [55]. Jasper also suggested that seizure discharge must affect the temporal lobes bilaterally in order to effect loss of consciousness, which he termed epileptic automatism with amnesia [36].

During primary and secondary generalized tonic-clonic seizures, the 10–20 second tonic phase of sustained muscle contractions with high-frequency EEG activity is followed by rhythmic limb contractions during clonic activity and a post-ictal phase. Patients are unconscious in that they are unresponsive to external stimuli and tend to have no recall of events. Rare exceptions have been reported, for example seizures beginning in central, extra-temporal brain regions close to motor cortex with limited effects outside the bilateral motor pathways possibly accounting for the tonic-clonic behavioural components of the seizure but consciousness, language, and memory processing were relatively unaffected [2].

Absence seizures are characterised by a brief loss of awareness and subtle motor changes with typical absence seizures electrographically associated with high amplitude 3 Hz spike-waves. Consciousness is usually transiently impaired [3].

Partial seizures may be classified further as simple partial seizures, with retained consciousness, or complex partial seizures, associated with a loss of consciousness. For seizures with a temporal lobe origin, simple partial seizures may include autonomic, psychic and epigastric symptoms. Complex partial seizures of temporal lobe origin often involve initial motor inhibition, ictal behavioural automatisms, unresponsiveness, postictal confusion and amnesia of the event. Although medial temporal lobe dysfunction is expected to cause memory loss it remains debatable why profoundly impaired consciousness is so common in temporal lobe seizures.

Outcomes from early hemispherectomy studies confirmed that a single hemisphere and intact brain stem connections were sufficient to sustain normal consciousness [1]. Hemispheric laterality of seizures did seem to influence consciousness. The left hemisphere was found to be usually responsible for auditory and verbal language skills whilst the right hemisphere related to awareness of corporeal and emotional self [16]. Left temporal lobe seizures have been reported to be more likely to generalise and associated with post-ictal aphasia compared to right temporal lobe seizures [20].

Inoue and Mihara [34] used intracranial monitoring in 142 presurgical subjects and assessed impaired consciousness as altered awareness and/or responsiveness. Bilateral seizure activity was correlated with impaired consciousness. Of the patients investigated, 33% ($n = 21$) of Frontal Lobe epilepsy, 25% ($n = 88$) of Temporal Lobe Epilepsy and 50% ($n = 4$) of Occipital Lobe Epilepsy responded to stimuli during seizures. On testing recall of the stimulus applied during seizures, 50% of Frontal Lobe Epilepsy patients could recall the stimulus (although only 25% responded to stimulus) but none of the other subjects could do so. Unilateral temporal seizure activity was found to be sufficient to impair consciousness assessed by altered awareness and/or responsiveness and was more likely to do so if activity was located in the lateral cortex of the language dominant temporal lobe or in the non-dominant frontal lobe but with extensive cortical involvement. Others have also correlated loss of consciousness in Temporal Lobe Epilepsy with bitemporal involvement e.g. [27].

However, evidence to support electrographic left temporal lobe lateralization of seizure activity as most likely to cause impaired consciousness may be dependent on the methods used to assess consciousness [23–47]. Many studies that have observed consciousness to be linked to the dominant hemisphere rely on verbal determinants of arousal to measure conscious response whereas measures of right temporal lobe function such as memory for faces or figures was not assessed. Additionally, unresponsiveness may be secondary to aphasia rather the loss of consciousness and inability to recall may be related to amnesia rather than impaired consciousness. The rarely used patient subjective accounts could provide useful additional information to observer-dependant assessments.

A Network Inhibition Hypothesis for loss of consciousness in epilepsy has been proposed [3,4,6,53]. According to this hypothesis absence, complex partial and tonic-clonic seizures share common neuroanatomical substrates for impaired consciousness. A proposed "consciousness system" consists of a number of structures including the medial thalamus, upper brain stem, interhemispheric regions (medial frontal cortex, cingulate, and precuneus), and lateral frontal and parietal association cortices. It is proposed that seizure activity propagates to the subcortical regions necessary for cortical activation, allowing the cortex to descend into an inhibited state of unconsciousness. Abnormal increased activity in the thalamus and upper brainstem disrupts the midline arousal systems and prevents normal activation of the cortex. This leads to abnormally reduced function in the fronto-parietal association cortex caused either by increased inhibitory inputs and/or decreased excitatory inputs resulting in impaired consciousness. Following most tonic-clonic and temporal lobe complex partial seizures, there is a postictal period with impaired consciousness, accompanied by decreased activity in the fronto-parietal association cortex. This postictal impairment is not present after most absence seizures.

The Network inhibition hypothesis is supported by observations that secondary generalised seizures demonstrating abnormal increases in thalamus, midbrain and cerebellum CBF during seizures are correlated with reduced CBF in fronto-parietal association cortex [7]. Human [5] and animal [18,19] electrophysiological studies also suggest limbic (hippocampal) seizures exert remote depressive effects on the neocortex observed as 1–2 Hz high amplitude slow waves which are considered to resemble coma, deep sleep or encephalopathy (rather than propagation of seizure activity) through disruption of midline subcortical networks. This abnormal decreased function in the fronto-parietal association cortex contrasts with fast polyspike activity and elevated cerebral blood flow in limbic and other subcortical structures ictally.

## 3. Neurostimulation

### 3.1. *Vagal nerve stimulation* (*VNS*)

The mechanisms of action of VNS are not fully understood. Theories have focused on the effects of VNS on cortical activity and the diffusely projecting nuclei of the brainstem such as the locus coeruleus, nucleus of the solitary tract (NST), thalamus and limbic structures. The brainstem nucleus of the solitary tract (NST) is the main relay station for afferent vagal nerve fibres. This nucleus has widespread projections to numerous areas in the forebrain, brainstem, thalamus and areas involved in learning and memory formation (amygdala, hippocampus) [30,70]. VNS induces fos immunoreactivity in the locus coeruleus and A5 noradrenergic nucleus [51]. In rats, a locus coeruleus lesion abolishes the beneficial effect of VNS [43]. Using a lateral fluid percussion injury (LFP) rat model of traumatic brain injury, VNS facilitated both the rate of recovery and the extent of motor and cognitive recovery [62]. In human studies, VNS has been found to increase (although not consistently) both catecholamine metabolites and GABA levels in CSF [10] and also influence synchrony across the cortex, enhancing gamma frequency synchrony whilst decreasing theta and delta synchrony [40, 48]. Whether this effect of VNS on hemispheric gamma synchrony is mediated by catecholamines is not known.

Learning, memory encoding and recall are modulated by arousal which is integral to most concepts of consciousness. Neurohormones such as ACTH, adrenaline, cortisol are associated with arousal and are thought to mediate the effect of arousal on memory. Many of the neurohormones that modulate memory do not freely cross the blood-brain barrier. VNS may enhance afferent vagal fibres may be involved in relaying information from peripheral receptors activated by neuromodulators to the CNS [18,31,48].

Hippocampal Long-Term Potentiation (LTP) is considered to underlie memory formation and involves a protein synthesis-independent early LTP lasting up to 4 or 6 hours and a protein synthesis-dependent late LTP that may last for days [44]. Using a rat model, the neural mechanisms underlying the mnemonic effect of VNS were associated with modulation of synaptic plasticity in the hippocampus through enhanced potentiation of the population spike amplitude for at least 24 hours using 0.4 mA biphasic stimulation and LTP induction with a weak tetanic stimulation delivered to the perforant path [72].

There are few human studies addressing cognition in epilepsy patients treated with VNS and no human studies specifically addressing the effects of VNS on consciousness.

Increased alertness in epilepsy patients with VNS implants has been reported more frequently in responders than non-responders [42,61]. However, this does not seem to influence memory. A small case-control study did not find any significant differences in objective memory scores using Wechsler Memory Scale III and the Memory Observation Questionnaire in VNS patients ($n = 16$) compared to those under medical management over 12 months ($n = 9$) [49]. Another small study ($n = 12$) investigating short-term memory in epilepsy patients using Mismatch Negativity latencies and amplitudes did not show significant changes 12 months after VNS implantation [8].

Chronic VNS has been shown not impair cognitive motor control [13]. In a small sample ($n = 5$) VNS was applied acutely during the consolidation process of the memory. VNS enhanced verbal memory maximally at 0.5 mA compared to sham VNS in this small epilepsy cohort [14]. The 0.5 mA maximal cognitive response for verbal memory is relatively low, for clinical use as a range of 1–2.5 mA is common. However, Hoppe et al [33] did not detect changes in attention, motor functioning, memory and executive function in in a larger study (n=36) of epilepsy patients before and after 6 months of VNS at standard clinical stimulation parameters. Contrary to the study of Clarke et al. [14] on verbal memory, Helmstaedter et al used 1–2.5 mA in an acute stimulation paradigm during both the presentation and recognition of words and designs. The study reported deterioration of figural memory, but not verbal memory. The deterioration was reversible. During chronic stimulation there was an improvement in decision times [32]. Type 2 error, different output currents and the timing of the acute exposure to VNS (memory consolidation as opposed to during learning and recognition) may explain the differing outcomes on memory tasks between the studies. Dodrill and Morris compared a high and low stimulation group of epilepsy patients after 3–4 months of VNS and found no statistically significant differences in cognitive tests [17]. In depressed patients treated with VNS, cognitive improvements have been reported but these are primarily in patients with clinical improvement in their mood disorder [57].

Although VNS is the only licensed neurostimulation technique in epilepsy, other technologies are under active research scrutiny. These will be discussed briefly in the context of consciousness.

### 3.2. *Deep brain stimulation* (*DBS*)

Although the cortex is considered the major influence on seizure generation, recent evidence has highlighted the role of subcortical structures in the expression, propagation and control of seizures [58,68]. DBS in epilepsy has been applied to a number of targets, including the thalamus (including the anterior and centromedian nuclei), cerebellum and basal ganglia (subthalamic nucleus, caudate, substantia nigra pars reticulata).

The thalamus, innervated by both the brainstem and basal forebrain arousal systems, is perhaps most relevant to neural circuits considered to underpin consciousness through distributed mechanisms of arousal regulation [21]. Focal brain injuries within the central thalamus can cause residual disturbances of consciousness if the lesions are bilateral due to extensive anatomical disconnection [11]. Neuroimaging studies demonstrate selective activation of the central thalamus for tasks that require a short-term attentional shift [71], sustained cognitive demands of high vigilance [54] and memory over extended time periods [11]. There is an active interest in central thalamic DBS in severe brain injury with the focus moving from chronically unresponsive patients who may not respond due to extensive disconnection to conscious patients with significant preservation of large-scale integrative cerebral networks [59].

The anterior nucleus of the thalamus is considered central in the network which underlies limbic seizures [15] and was the site of stimulation subject to a multicentre DBS trial (SANTE) for medically refractory focal-onset seizures. Bilateral anterior nucleus stimulation did not demonstrate any difference for cognition and mood between control and stimulated groups at the end of the 3 month blinded phase of the DBS SANTE trail [22]. The centromedian nucleus of the thalamus is involved in the reticulothalamocortical system mediating cerebral cortex excitability and has been considered integral to the modulation of vigilance [37, 66]. However, there is little data on the specific effects of DBS in epilepsy in relation to consciousness and cognition. In a small pilot study ($n = 7$) of bilateral centromedian thalamic DBS, stimulation at low intensity did not alter the EEG acutely. However, high-intensity stimulation induced slow waves or 2–3 Hz spike-waves. There were no DBS-related changes in cognitive function [22]. Centromedian nucleus DBS has also been utilised in movement disorders. Bilateral DBS in the centromedian-parafascicular and ventralis oralis complex of the thalamus in 15 subjects with Tourette syndrome did not reveal differences on measures of cognitive functions before and after DBS at 24 month follow-up [56].

Acute unilateral DBS in the globus pallidus internus (GPi) for cervical dystonia under general anaesthetic was reported to have caused a transient state of wakefulness proportional to stimulus intensity with eye opening in response to verbal command or external stimuli but without detectable signs of conscious awareness. The EEG was in the gamma frequency range (40–120 Hz) bilaterally and associated with autonomic arousal. These effects were observed for multiple anatomical loci between the GPi and Nucleus Basalis of Meynert. This sub-pallidal DBS is thought to have reversed an anaesthesia-induced inhibition by direct excitation of an arousal circuit or inhibition of a sleep-promoting centre in the basal forebrain [50].

### 3.3. Direct cortical stimulation

Stimulation at the seizure focus using repetitive transcranial magnetic stimulation (rTMS) or invasive cortical stimulation is also under active investigation.

In humans, low frequency rTMS reduces motor cortex excitability, while high frequency rTMS can lead to seizures [12]. rTMS is more likely to be effective in patients with clearly identifiable foci in the cortical convexity [24] and some preliminary cognitive benefits were observed in the active group in this study. Cognitive improvement after high-frequency stimulation specifically over the left dorsolateral prefrontal cortex has been reported in heterogeneous populations (psychiatric, neurological and healthy volunteers) although the mechanisms are poorly understood [31].

Direct invasive stimulation of epileptic foci has also shown potential for an antiepileptic effect [64,67]. Adaptive stimulation has advantages in using seizure-triggered stimulation rather than a continuous duty cycle and is encompassed in The Responsive Neurostimulation System (NeuroPace) using intracranial electrodes in situ to continuously monitor brain electrical activity and complex algorithms to identify seizure onset and deliver brief electrical stimulation to suppress the seizure. The influence of direct cortical stimulation on specific aspects of consciousness in epilepsy subjects is not known.

## 4. Conclusions

Consciousness is often disrupted in epilepsy. The Network Inhibition Hypothesis proposes a common neuroanatomical substrate for impaired consciousness during absence, complex partial and tonic-clonic seizures.

Neurostimulation in epilepsy remains in its infancy with vagal nerve stimulation (VNS) as the only firmly established technique and a series of other methods under investigation including deep brain stimulation (DBS), intracranial cortical stimulation and repetitive transcranial magnetic stimulation (rTMS). Many of these systems impact on the neural systems thought to be involved in consciousness as a continuous duty cycle although some adaptive (seizure triggered) techniques have been developed.

Theoretically, fixed duty cycle neurostimulation could have profound effects on aspects of consciousness including alertness, responsiveness, and awareness of self and subjective experience. Animal studies suggest vagal nerve stimulation positively influences hippocampal long term potentiation. In humans, increased alertness in VNS implanted subjects and an acute effect on memory consolidation has been reported but convincing data on either improvements or deterioration in attention and memory is lacking. Neither bilateral anterior thalamic nucleus nor centromedian thalamic nucleus DBS seem to affect cognition.

As intracranial neurostimulation, particularly thalamic DBS, becomes more established as a clinical intervention, the effects on consciousness and cognition with variations in stimulus parameters will need to be studied to understand whether these secondary effects of neurostimulation make a significant positive (or adverse) contribution to quality of life in epilepsy.
